# Acute Myocardial Injury in a Patient with Attention Deficit Hyperactivity Disorder and History of Substance Abuse: A Multimodality Imaging Point of View

**DOI:** 10.3390/jcdd8060067

**Published:** 2021-06-07

**Authors:** Sahrai Saeed, Svein Rotevatn, Jan Schjøtt, Terje H. Larsen

**Affiliations:** 1 Department of Heart Disease, Haukeland University Hospital, 5021 Bergen, Norway; svein.rotevatn@helse-bergen.no (S.R.); terje.hjalmar.larsen@helse-bergen.no (T.H.L.); 2 Department of Clinical Biochemistry and Pharmacology, Haukeland University Hospital, 5021 Bergen, Norway; jan.didrik.schjott@helse-bergen.no; 3 Department of Clinical Science, Faculty of Medicine, University of Bergen, 5021 Bergen, Norway

**Keywords:** amphetamine, attention deficit hyperactivity disorder, cannabis, cardiac magnetic resonance, echocardiography, ST-segment elevation myocardial infarction, substance abuse

## Abstract

Both cannabis and amphetamine are the most commonly used illegal substances worldwide and are associated with a number of adverse cardiovascular effects including transient coronary vasospasm. Here, we present the case of a 39-year-old male admitted to our institution with a 6-h history of severe chest pain and ST-segment elevation on the ECG. Coronary angiography on admission showed normal coronary arteries. The patient had a 14-year history of substance abuse, primarily amphetamine and cannabis, and was prescribed lisdexamfetamin (Aduvanz^®^) for attention deficit hyperactivity disorder (ADHD) for the past 2 years. A cardiac magnetic resonance (CMR) the following day showed widely distributed focal lesions of late gadolinium enhancement in mid- and sub-epicardial myocardium in the anterior, lateral and inferior walls, suggestive of chronic fibrotic lesions. There was no sign of acute myocardial edema. No viral cause was identified during a thorough investigation, including negative SARS-COV-2 and endomyocardial biopsy. Substance-abuse-induced coronary vasospasm leading to ST-segment elevation, myocardial damage with a rise and fall of cardiac TnT, as well as a slightly reduced left ventricular ejection fraction (48%) and regional wall motion abnormalities on echocardiography, was the most likely diagnosis.

## 1. Introduction

Both cannabis and amphetamine are the most commonly used illegal substances worldwide and are associated with a number of adverse cardiovascular effects including ST-segment elevation myocardial infarction due to acute coronary vasospasm, and cardiomyopathies due to chronic toxic effect on the myocardium [1,2]. In the case of acute myocardial injury due to acute coronary vasospasm, coronary angiography may typically show normal coronary anatomy. 

## 2. Case Presentation

**Figure 1:**
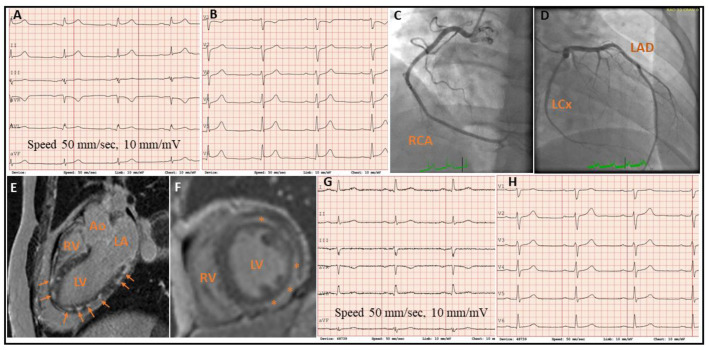
The electrocardiogram (**A**,**B**) on admission showing 1 mm ST-segment elevation in leads I, AVL and V5–V6, and 1 mm ST-segment depression in lead V1. Coronary angiography (**C**,**D**) shows normal RCA, LCx and LAD. The cardiac magnetic resonance images (**E**,**F**) demonstrate dispersed focal lesions with LGE in mid- and sub-epicardial myocardium (arrows and asterisks). The electrocardiogram at discharge (**G**,**H**) displays normalization of the ST-segments. Ao, aorta; LA, left atrium; LAD, left anterior descending artery; LCx, left circumflex artery; LGE, late gadolinium enhancement; LV, left ventricle; RCA, right coronary artery; RV, right ventricle.

A 39-year-old Norwegian male was admitted to our department with a 6-h history of severe chest pain and minor ST-segment elevations on ECG (Figure 1A,B).

**Figure 2:**
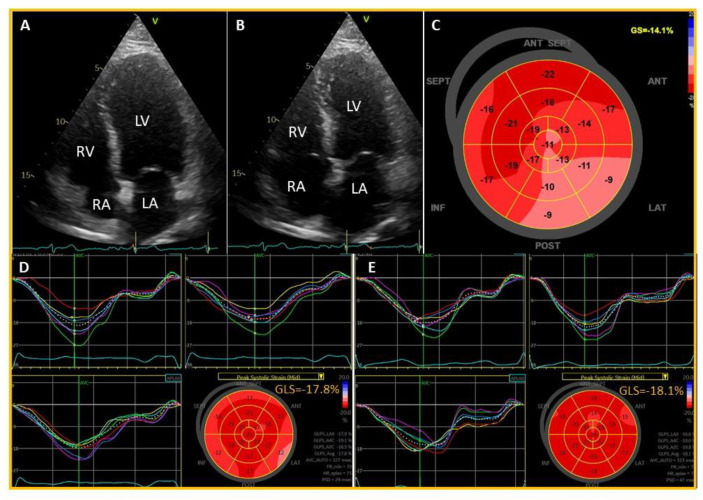
Echocardiographic images of the heart on admission (**A**–**C**), at discharge (**D**) and at 6-weeks follow-up (**E**). (**A**) demonstrates end-diastolic and (**B**) end-systolic frames on apical four chamber views. The distal septal segments towards the apex are hypokinetic and slightly dilated (**A**) and shows little motion toward the mid-cavity during end-systole (**B**). The Bulls eye plot shows reduced (less negative) strain values in lateral and posterior walls from basal to apical segments (**C**) with an average global longitudinal strain (GLS) of −14.1%. (**D**) displays improvement of GLS to −17.8% on discharge but still some impaired strain value in a basal segment of the lateral wall. Strain values were further improved to −18.1% at 6-weeks follow-up (**E**), although there was some post-systolic shortening (left upper and lower panels). LA, left atrium; LV, left ventricle; RA, right atrium; RV, right ventricle.

He had a 14-year history of substance abuse, primarily amphetamine and cannabis, and was prescribed lisdexamfetamin (Aduvanz^®^) for attention deficit hyperactivity disorder (ADHD) for the past 2 years. He was a non-smoker, had no previous history of diabetes or hypertension and had a BMI of 33 kg/m^2^. On admission, the heart rate was 65 beats per minute and the blood pressure 123/78 mmHg. Urgent coronary angiography revealed normal epicardial coronary arteries (Figure 1C,D). Cardiac troponin T (TnT) was elevated at 319 ng/L (normal range < 16 ng/L) on admission which further increased to 1148 ng/L four hours later, and 2035 ng/L the next day, before it declined to 19 ng/L at discharge. Pro-BNP was 420 ng/L (normal range < 85 ng/L) on admission, 2648 ng/L the next day and 992 ng/L on the day before discharge. The echocardiogram on admission showed normal left ventricular (LV) wall thickness and dimensions, and mildly reduced LV ejection fraction (48%) with hypokinesis in the apex, and in the lateral and inferior wall distal segments towards the apex associated with reduced strain in the same segments (Figure 2A–C).

A cardiac magnetic resonance (CMR) the following day showed widespread, focal lesions of late gadolinium enhancement (LGE) in the mid- and sub-epicardial myocardium in the anterior, lateral and inferior walls, suggestive of chronic replacement fibrosis (Figure 1E,F). LV ejection fraction was 46% with hypokinesis of the inferolateral myocardial segments. There was no sign of myocardial edema. Cardiotrophic viruses and SARS-COV-2 were negative, and an endomyocardial biopsy did not show any sign of common viral or autoimmune myocarditis. The ECG at discharge showed complete resolution of ST-segment elevations (Figure 1G,H). Substance-abuse-induced coronary vasospasm leading to a typical ST-segment elevation on the ECG and myocardial damage with a typical rise and fall of cardiac TnT was the most likely diagnosis. No anamnestic information about recent substance abuse was available when the patient was admitted to hospital. Hence, the triggering factor for an acute ST-segment elevation myocardial infarction in the context of normal epicardial coronary arteries remained uncertain at this point. The echocardiography at discharge showed normalization of LV ejection fraction (55%) and no sign of regional wall motion abnormalities on Bulls eye plot (global longitudinal strain [GLS] −17.8%), apart from reduced strain in the basal lateral segment of −12% (Figure 2D), which remained stable at 6-week follow-up although there was still some post-systolic shortening in some segments on the apical 4- and 3-chamber views (Figure 2E). Upon a repeated and careful history taking at 6-weeks follow-up the patient admitted that during the weekend, 24-h prior to his hospitalization, he had consumed amphetamine as a sniffing inhalant, which was probably the trigger for the index event. The patient had a similar hospitalization with acute chest pain which occurred at mid-night, with ST-segment elevation on the ECG and a raised TnT level, with normal coronary arteries on angiography 14-years ago. The echocardiogram had shown slightly reduced LV systolic function with LV ejection fraction of 45%–50%, and hypokinesis in the anterior wall, which had reversed by discharge a week later. A CMR three days later had not revealed any sign of acute myocarditis or myocardial edema (data not shown). All viral PCR tests were negative. The condition was concluded as stress-cardiomyopathy (Takotsubo syndrome), but myopericarditis was a possible differential diagnosis. The emerging new substance abuse history went initially undetected when the patient was admitted to hospital, and acute cardiac injury by severe coronary vasospasm due to the toxic effects of substance abuse were not suspected at the time. Thus, CMR was not performed immediate after admission which could have possibly diagnosed stress cardiomyopathy from infarct-pattern LGE within the myocardium. 

## 3. Discussion

This case report has two important learning points: First, there were two episodes of acute cardiac injury manifested by severe chest pain, ST-segment elevation on the ECG, transient LV dysfunction on echocardiography, elevated cardiomyocyte-specific biomarkers and angiographically normal epicardial coronary arteries, which occurred 14-years apart, but both episodes occurred at midnight while the patient was supposedly sleeping. The cause of the first episode was uncertain and Takotsubo syndrome was suspected, though the patient did not report any specific trigger/stress factor. Myocarditis was an alternative differential diagnosis. However, the CMR three days later showed normal LV myocardium. Most acute changes such as wall motional abnormalities and LV dysfunction, as well as myocardial edema in the case of acute transient cardiac injury may reverse to normal within a few days following the episode of acute Takotsubo or a mild self-terminating myocarditis. The history of cannabis and amphetamine abuse went on over a period of 14 years, which caused extensive toxic effects on the LV myocardium with distinct CMR features of chronic fibrotic lesions: multiple focal hyperintensity signals (LGE) in inferior, lateral and anterior walls. 

Cannabis and amphetamine are the most commonly used illegal substances worldwide [1,2]. The acute cardiovascular complications of amphetamine reported in the literature are among others tachycardia, elevated blood pressure, myocardial infarction, coronary vasospasm, arrhythmias, stress cardiomyopathy and recurrent myopericarditis [1,2,3,4,5,6,7]. Further, the chronic adverse cardiovascular effects of amphetamine may be pulmonary hypertension, atherosclerotic plaque formation and indications of cardiomyopathy (necrosis, fibrosis) on CMR with or without functional and other structural remodeling of the heart [8].

In case reports on cannabis- and amphetamine-induced acute coronary syndromes with typical ST-segment elevation on ECG, normal epicardial coronary arteries were described on coronary angiography and the events were attributed to transient coronary vasospasm [2,5]. This was also most likely the case in our patient. The duration of ischemia due to acute coronary vasospasm was probably short and the CMR did not show signs of acute myocardial edema on T2-weighted imaging. Nevertheless, the CMR-findings were strongly indicative of a chronic myocardial toxicity with extensive patchy pattern distribution of LGE within the myocardium due to chronic cannabis as well as amphetamine abuse. Furthermore, LV dysfunction and regional wall motion abnormalities disappeared within a few days and LV function was almost normal at discharge, although there was still some impairment of regional strain values. At 6-weeks follow-up, LV ejection fraction and global and segment strain values had returned to normal. However, some degree of post-systolic shortening was still evident. Post-systolic shortening, defined as shortening in diastole beyond minimum systolic length, is examined in patients with acute myocardial infarction [9], but its clinical significance in acute coronary vasospasm induced by substance abuse is not explored. 

Second, our patient had ADHD and was treated with a high dose of lisdexamfetamine (70 mg) (Aduvanz^®^). Lisedexamfetamine is a prodrug that is rapidly converted to dexamfetamine in blood with a half-life of 1 h, while dexamfetamine has a half-life of about 11 h [10]. Sniff inhalation of amphetamine 24-h prior to his hospitalization might have resulted in additive adverse cardiovascular effects, and led to acute coronary vasospasm with subsequent cardiac injury. Most importantly, amphetamine-induced acute coronary spasm in ADHD patients treated with lisdexamfetamine should be borne in mind. Combined use of a prescribed amphetamine and illicit amphetamine could result in acute toxic effects due to overdose. 

The cardiovascular complications of psychostimulant drugs such as methylphenidate (Ritalin^®^) and lisdexamfetamine (Aduvanz^®^) in adults with ADHD are less studied [11]. However, some case reports have documented an association between these drugs and acute coronary syndromes in the context of angiographically normal coronary arteries, and myocarditis and cardiomyopathy [12,13]. Acute coronary syndromes in these patients may occur due to either coronary vasospasm or increased oxygen-demand mismatch secondary to tachycardia, shortening of diastole and sub-endocardial ischemia, as well as increased arterial load due to systemic hypertension and increased sympathetic nerve activity [11,12,13]. The latter can be further modified or increased by ADHD, as well as stimulant medications for the disease. 

When taking patient history, special attention should be aimed at gathering information of exposure to illicit drugs or toxic substances among others misuse of cannabis, cocaine and amphetamine, as well as therapeutic use of ADHD medications in high doses such as methylphenidate and lisdexamfetamin. Most importantly, amphetamine-induced acute coronary spasm in ADHD patients treated with lisdexamfetamine should be borne in mind as the combination entails risk of overdose. 

## 4. Conclusions

Amphetamine is one of the most commonly used illicit substances and is associated with a wide range of serious cardiovascular complications, including ST-segment elevation myocardial infarction due to acute coronary vasospasm, and cardiomyopathies due to chronic toxic effect on the myocardium, characterized as patchy pattern myocardial damage/fibrosis. Our case shows that the risk of amphetamine-induced acute coronary spasm in ADHD patients treated with lisdexamfetamine should be borne in mind. 

## Data Availability

Upon a reasonable request, the authors will make the data available to any researcher for purposes of reproducing the results or replicating the procedure.
